# Bilateral adrenal haemorrhage secondary to intra-abdominal sepsis: a case report

**DOI:** 10.4076/1757-1626-2-6894

**Published:** 2009-06-09

**Authors:** Aoife M Egan, John O Larkin, Ronan S Ryan, Ronan Waldron

**Affiliations:** 1 Department of Surgery, Mayo General HospitalCastlebar, Co. MayoIreland; 2 Department of Radiology, Mayo General HospitalCastlebar, Co. MayoIreland

## Abstract

**Introduction:**

Bilateral adrenal haemorrhage is a rare cause of adrenal failure. Clinical features are non-specific and therefore a high index of suspicion must be maintained in patients at risk. Predisposing factors include infection, malignancy and the post-operative state.

**Case presentation:**

We report the case of a patient who underwent a left hemicolectomy with primary anastomosis and formation of a defunctioning loop ileostomy for an obstructing colon carcinoma at the splenic flexure. En-bloc splenectomy was performed to ensure an oncologic resection. The patient developed a purulent abdominal collection post-operatively and became septic with hypotension and pyrexia. This precipitated acute bilateral adrenal haemorrhage with consequent adrenal insufficiency. Clinical suspicion was confirmed by radiological findings and a co-syntropin test. Following drainage of the collection, antibiotic therapy and corticosteroid replacement, the patient made an excellent recovery.

**Conclusion:**

This case highlights the importance of prompt diagnosis and treatment of adrenal failure. In their absence, this condition can rapidly lead to death of the patient.

## Introduction

Bilateral adrenal haemorrhage is rarely diagnosed clinically as its presentation is generally non-specific. The clinical importance of bilateral adrenal haemorrhage is that it may lead to acute adrenal insufficiency and possible death [1]. We present the case of an elderly male patient with acute adrenal insufficiency due to bilateral adrenal haemorrhage following an emergency bowel resection and en-bloc splenectomy complicated by intra-abdominal sepsis.

## Case presentation

An 81-year-old man presented to our institution with a ten day history of constipation with progressively worsening lower abdominal pain, nausea and vomiting. His past medical history was remarkable only for uncomplicated peptic ulcer disease for which he was on maintenance ranitidine. On examination, he was distressed and dehydrated with normal vital signs. The abdomen was markedly distended with tenderness in the left lower quadrant. The rectum was empty. Haemoglobin was 13.9 g/dL, white cell count was 11.5 × 109/L, serum sodium was 133 mmol/L and potassium 3.9 mmol/L. A plain film of the abdomen demonstrated a distended transverse colon with no air in the rectum. The working clinical diagnosis was complete large bowel obstruction and so the patient was resuscitated with intravenous fluids and commenced on nasogastric decompression and intravenous proton pump inhibitor therapy.

On-table colonoscopy confirmed an obstructing tumour at the splenic flexure. A number of bilobar metastatic lesions were noted in the liver at laparotomy. A left hemicolectomy with primary anastomosis and formation of a defunctioning loop ileostomy was performed along with an en-bloc splenectomy. It was necessary to remove the spleen due to local tumour invasion in order to ensure an oncologic resection. Histopathological analysis of the resected specimen revealed an adenocarcinoma of the colon.

The patient improved clinically until post-operative day six when a rise in CRP (150 mg/l) and white cell count (20 × 10^9^/l) was observed. His temperature rose to 38.5^o^C. On day eight, the patient's condition deteriorated acutely and he became hypotensive (bp: 86/50 mmHg). This necessitated a noradrenaline infusion, with the dose ranging from 3-8 µg/kg/hr to maintain a satisfactory mean arterial pressure. An abdominal ultrasound demonstrated and simultaneously guided drainage of a purulent collection lateral to the left kidney. He had been receiving prophylactic intravenous penicillin in light of his asplenic state, but at this stage, intravenous piperacillin/tazobactam and gentamicin were commenced in light of the ultrasound findings. Enterococcus faecalis was subsequently isolated from the aspirated pus.

**Figure 1.**
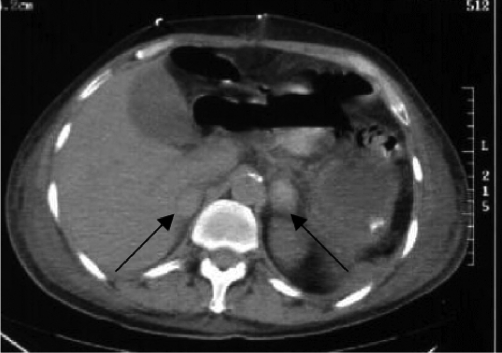
CT scan of the abdomen and pelvis. Arrows demonstrate bilateral enlargement and hyperdensity of the adrenal glands consistent with adrenal haemorrhage.

A subsequent Computed Tomography (CT) scan of the abdomen and pelvis (performed without intravenous contrast due to marked transient renal impairment) demonstrated bilateral enlargement and hyperdensity of the adrenal glands consistent with adrenal haemorrhage (Figure 1). Serum sodium was 130 mmol/L, potassium was 5.1 mmol/L and glucose was 3.8 mmol/L. A diagnosis of adrenal insufficiency was therefore made and 100 mg of hydrocortisone was administered intravenously. Seven hours after commencement of steroid therapy the noradrenaline infusion was discontinued as the patient's blood pressure had stabilised. He was transferred to the ward on post-operative day ten and the hydrocortisone was changed to oral administration.

**Figure 2.**
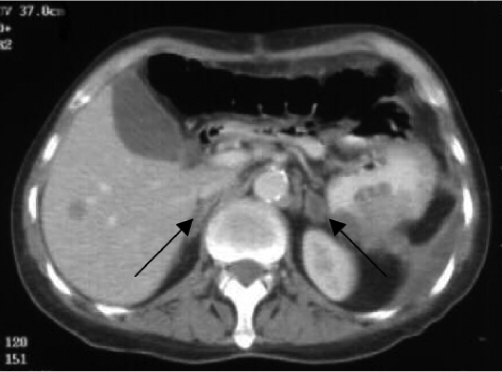
Repeat CT scan of thorax, abdomen and pelvis, five weeks after first scan. Arrows demonstrate interval liquefaction of the adrenal gland haemorrhages.

Prior to discharge from hospital, hydrocortisone was stopped for 24 hours and a cosyntropin stimulation test was performed using 250 µg of cosyntropin. This demonstrated an inadequate response to adrenocorticotropic hormone (ACTH) - serum cortisol at 30 and 60 minutes post ACTH was 10.2 µg/dl and 10.1 µg/dl respectively. The patient was discharged from hospital on a maintenance dose of oral hydrocortisone and was treated with a palliative course of chemotherapy. A repeat CT scan of thorax, abdomen and pelvis some five weeks later (with intravenous contrast, renal function having returned to normal) demonstrated metastatic lung and liver lesions and interval liquefaction of the adrenal gland haemorrhages (Figure 2). He continues to take oral hydrocortisone, is on lifelong penicillin prophylactically and remains clinically well at out-patient follow-up six months later.

## Discussion

The adrenal glands are of intrinsic importance in the physiological response to stress, responsible for maintenance of blood pressure and electrolyte homeostasis [2]. In critically ill patients, unrecognised and untreated adrenal insufficiency is usually fatal [2] and as the presentation of acute adrenal insufficiency is variable and non-specific, a high degree of clinical suspicion is required.

Clinical features associated with adrenal insufficiency caused by adrenal haemorrhage include pain, fever, nausea, vomiting, fatigue, weakness, obtundation, anorexia, and hypotension. Laboratory features include hyponatraemia, hyperkalaemia, uraemia, hypoglycaemia, eosinophilia and anaemia. The clinical and laboratory features may or may not all be present, and thus it is extremely important to remain alert to the possibility of this diagnosis in patients at risk for developing adrenal haemorrhage [3,4].

Bilateral adrenal haemorrhage is a rare cause of acute adrenal failure, generally occurring in hospitalised patients who are septic, coagulopathic or who have thromboembolic disorders. The post-operative or post-myocardial infarction states are risk factors. Adrenal haemorrhage secondary to sepsis most typically occurs in the presence of meningococcaemia and is referred to as Waterhouse-Friederichsen Syndrome. There is increasing evidence of hypothalamic-pituitary-adrenal insufficiency in critically ill septic patients which appears to result from circulating suppressive factors released during systemic inflammation. This is associated with both primary and secondary adrenal insufficiency [5]. The prevalence of adrenal insufficiency in septic shock varies but is estimated to occur in approximately half of all septic shock cases [4,5].

The pathogenesis of adrenal haemorrhage is typically multifactorial. The adrenal gland receives a rich blood supply from the aorta and the inferior phrenic and adrenal arteries, which form a subcapsular plexus. Necrosis and haemorrhage may occur during hypotension and stress as a result of ischemia or during adrenal stimulation from vascular engorgement and stasis [6-8]. Ligation of the right adrenal vein during orthotopic liver transplantation is a recognised cause of adrenal infarction leading to haemorrhage [9].

Platelet aggregation followed by venous thrombosis, vasoconstriction, disruption of the vascular endothelium and regional hypotension are major contributors to the pathogenesis of adrenal haemorrhage, all of which lead to intramedullary ischaemia. Bleeding ensues with reperfusion of these damaged vessels [10].

The diagnosis of adrenal insufficiency resulting from adrenal haemorrhage is often overlooked because of the nonspecific nature of the clinical presentation. Until recently, most diagnoses of adrenal haemorrhage were made at postmortem examination. The reported incidence of adrenal haemorrhage in general hospital autopsy studies is 0.14% to 1.8% [11].

Adrenal insufficiency may be established in patients with a random serum cortisol level [4]. This condition can be excluded if the serum cortisol is >34 μg/dL. Conversely, the diagnosis is likely if the serum cortisol is <15 μg/dL. After intravenous administration of 250 μg of cosyntropin, a serum cortisol value at 30 or 60 minutes later of <14.9 μg/dL also points towards adrenal insufficiency [12]. Before testing, dexamethasone can be used as corticosteroid replacement because it is not measured by the cortisol assay.

The criteria for biochemical diagnosis of adrenal insufficiency in the critically ill patient remain unclear and are controversial. Some critically ill patients produce inappropriately low amounts of cortisol in relation to their needs because of the severity of their disease, even with apparently normal basal serum cortisol levels, so called relative adrenal insufficiency [2]. In these patients, a simultaneous measurement of serum cortisol and corticotrophin level is useful. The serum cortisol level will be inappropriately low in relation to the serum corticotrophin level. Of course, while corticotrophin stimulation is helpful in the diagnosis, it is not practical in patients with impending or established shock. In practice, a decrease in inotropic requirement and clinical improvement following administration of corticosteroids suggests adrenal insufficiency.

Ultrasound, CT, and magnetic resonance imaging (MRI) can all be used to suggest and/or confirm the diagnosis of adrenal haemorrhage, but CT is probably most useful and easily performed in critically ill patients. A hyperdense mass seen on non-intravenous contrast-enhanced CT scanning with no enhancement after intravenous contrast or enhancement only in a pattern of a thin peripheral rim can be valuable in discriminating a haematoma from adrenal tumours [13].

The treatment of adrenal insufficiency consists of prompt administration of parenteral hydrocortisone, tapered to maintenance oral hydrocortisone once the acute illness resolves [4]. In primary adrenal insufficiency, mineralocorticoids can be added once the dose of hydrocortisone is <50 mg daily, however in a published case series, prolonged mineralocorticoid replacement was usually not required in patients with adrenal insufficiency due to bilateral adrenal haemorrhage [14].

## Conclusion

We report the case of an elderly male patient who had a colectomy with en-bloc splenectomy for an obstructing colon carcinoma who developed a purulent abdominal collection post-operatively. The subsequent septic state precipitated acute bilateral adrenal haemorrhage with consequent adrenal insufficiency. CT scanning and biochemical profile including a cosyntropin test confirmed the diagnosis. Following radiological image-guided drainage of the purulent abdominal collection, aggressive antibiotic therapy and corticosteroid replacement, the patient made a good recovery.

Bilateral adrenal haemorrhage is a rare cause of adrenal insufficiency and typically presents with nonspecific clinical features. Thus, a high index of clinical suspicion must be maintained for patients with risk factors. Early diagnosis and corticosteroid replacement with aggressive management of the precipitating pathology are essential to enable a successful outcome.
